# Comparative assessment of canine-origin *Lactobacillus johnsonii* CPN23 and dairy-origin *Lactobacillus acidophillus* NCDC 15 for nutrient digestibility, faecal fermentative metabolites and selected gut health indices in dogs

**DOI:** 10.1017/jns.2017.35

**Published:** 2017-07-31

**Authors:** Sachin Kumar, Ashok K. Pattanaik, Shalini Sharma, Reema Gupta, Sunil E. Jadhav, Narayan Dutta

**Affiliations:** Niche Area of Excellence in Clinical Nutrition, Division of Animal Nutrition, ICAR Indian Veterinary Research Institute, Izatnagar-243 122, India

**Keywords:** Canine nutrition, Faecal metabolites, *Lactobacillus*, Species-specific probiotics, BCFA, branched-chain fatty acid, CMI, cell-mediated immunity, cPRO, probiotic of canine origin (*Lactobacillus johnsonii* CPN23), dPRO, probiotic of dairy origin (*Lactobacillus acidophilus* NCDC 15), DTH, delayed-type hypersensitivity

## Abstract

The present experiment was undertaken to validate a probiotic of canine origin for its potential use in dogs. A total of fifteen adult female Labrador dogs were allocated to three equal groups and fed a basal diet without probiotic (control) or with probiotic of either canine (*Lactobacillus johnsonii* CPN23; cPRO) or dairy (*L. acidophilus* NCDC 15; dPRO) origin for 9 weeks. The digestibility of most macronutrients remained similar among the groups; however, fibre digestibility was improved (*P* = 0·034) in dogs receiving cPRO. The faecal fermentative metabolites ammonia (*P* *<* 0·05) and lactate (*P* = 0·094) were altered favourably, indicating a positive influence of both probiotics. Faecal concentrations of acetate, propionate and butyrate were increased (*P* *<* 0·01) in both probiotic groups. However, improvements were higher in cPRO *v*. dPRO. The delayed-type hypersensitivity reaction to intradermal inoculation of phytohaemagglutinin-P was higher (*P* = 0·053) in cPRO as compared with control. The antibody response to sheep erythrocytes was, however, similar across the three groups. Overall, in dogs, the canine-origin probiotic was superior when compared with the dairy-origin probiotic.

Probiotics are micro-organisms that are added to the diet to exert beneficial effects on the host. One important criterion for selection of a probiotic is the host species specificity^(^^1^^)^. It is believed that probiotic organisms should be naturally occurring in the target species to be effective^(^^2^^)^ by inducing a greater production of SCFA in the hindgut and effecting optimal mucosal immunity. SCFA, in turn, play important health-promoting roles in the maintenance of gut barrier function by contributing to energy needs of host cells, absorption of select nutrients and inhibition of pathogenic micro-organisms^(^^3^^)^. Probiotics have the potential to stimulate innate immune responses without inducing inflammation; they interact with dendritic cells and follicle-associated epithelial cells and initiate responses mediated by macrophages and T- and B-lymphocytes^(^^4^^)^.

Adhesion of probiotic bacteria to epithelial cells is host specific; hence, for improved colonisation, the probiotic bacteria should originate from the same host species^(^^5^^)^. Commensal organisms may exert species-specific effects, and therefore, a successful canine probiotic organism would ideally be derived from the canine gastrointestinal tract^(^^6^^)^. However, there are only a few studies on canine-sourced bacteria as probiotics for dogs^(^^7^^,^^8^^)^.

Further, the available literature shows that most of the probiotics studies in dogs have been carried out with extruded pet foods as the basal diet. In most developing countries, including India, pet dogs are reared mainly on home-cooked diets of varied composition. However, few studies have examined the effects of probiotics when supplemented with homemade vegetarian diets. The present study, therefore, evaluated the potential of a canine-origin probiotic *v*. a dairy-origin probiotic for its possible use in dogs fed a homemade vegetarian diet.

## Materials and methods

### Animals, housing and management

The study protocol was approved by the Institutional Animal Ethics Committee, and was carried out in conformity with Committee for the Purpose of Control and Supervision of Experiments on Animals (CPCSEA) guidelines. Dogs used for the study were housed under hygienic conditions in a well-ventilated kennel having separate cubicles (1·52 × 0·91 m^2^) for individual housing and care. The dogs were let out in an open space adjacent to the kennel in the morning and evening for exercise and socialisation except during the collection period.

### Animals and diets

Fifteen adult female Labrador dogs (aged about 5 years; 22·9 (se 0·6) kg average body weight) were randomly allocated to three equal groups based on body weight and fed a pressure-cooked moist diet. The dogs had an ideal body condition score and were declared healthy by a veterinarian based on medical history, physical examination, complete blood count and serum biochemistry. The basal diet was specially formulated to meet National Research Council recommendations (adequate intake) for adult maintenance^(^^9^^)^ (Supplementary Table S1). The amount of food was calculated to meet the maintenance energy requirement (kcal = 130 × kg body weight^0·75^; kJ = 544 × kg body weight^0·75^)^(^^9^^)^. The diet was supplemented with either no probiotics (control), or with a probiotic of canine origin (*Lactobacillus johnsonii* CPN23; cPRO) or dairy origin (*Lactobacillus acidophilus* NCDC 15; dPRO). The control group received a placebo (De Man, Rogosa and Sharpe broth), while cPRO and dPRO groups received cultures of the respective probiotics (at 2·3 × 10^8^ colony-forming units/animal per d) mixed with the basal diet.

The canine-origin probiotic *L. johnsonii* CPN23 was previously developed in our laboratory and characterised using 16S rRNA analyses (GenBank accession no. KP065494). A freeze-dried pure culture of the *L. acidophilus* NCDC 15 strain, procured from the National Collection of Dairy Culture (NCDC), National Dairy Research Institute (Karnal, India) was used for feeding the dPRO group.

All the dogs had 24 h access to clean and fresh water *ad libitum*. Before the study, dogs were adapted to the basal diet for a period of 15 d. Each individual dog's ration was divided into two equal portions and fed once each in the morning (09.00 hours) and evening (17.00 hours). Probiotics were administered only in the morning. The experimental period lasted for 9 weeks.

### Experimental protocol

Food consumption was monitoring daily. A 4 d digestibility trial was conducted after 7 weeks of feeding as described earlier^(^^10^^)^ to calculate the apparent digestibility (nutrient intake – nutrient output/nutrient intake × 100). A 1–4-point palatability score was adopted for subjective assessment of the acceptability of the experimental diets^(^^11^^)^. The faeces voided by individual dogs, collected quantitatively over the preceding 24 h, were weighed individually for each dog, mixed thoroughly and used for further sampling and analysis. The faecal consistency score was recorded based on a 1–5-point scale^(^^11^^)^. Three different aliquots were drawn from the faecal samples and processed for the determination of DM, N and fermentative metabolites as described elsewhere^(^^10^^)^. The samples of faeces and food were dried at 60°C in a forced-draft oven, ground through a 2 mm screen in a laboratory mill (SM100; Retsch GmbH) and stored in airtight high-density polyethylene jars pending further analysis.

### Laboratory analyses

The ground samples of food and faeces were analysed for DM, organic matter, crude protein, ether extract, crude fibre and crude ash, while N-free extract was calculated^(^^12^^)^. The pH of the faecal samples was determined by a pH meter (Eutech Instruments). Lactate, ammonia, SCFA and branched-chain fatty acid (BCFA) concentrations in the faecal samples were analysed as described earlier^(^^13^^)^.

Cell-mediated immunity (CMI) was assessed at 8 weeks by measurement of skin indurations as type-IV delayed-type hypersensitivity (DTH) reaction to phytohaemagglutinin-P (Sigma) as a mitogen, as detailed previously^(^^14^^)^. For the humoral immune response, dogs were intravenously injected with 1 ml of a 10 % suspension of washed sheep erythrocytes after 5 weeks of experimental feeding. Serum samples collected at periodic intervals (0, 7, 14, 21 and 28 d) were used for an antibody titre assay using the microtitre haemagglutination procedure^(^^15^^)^.

### Statistics

The data were analysed by one-way ANOVA using SPSS 20.0 (SPSS Inc.). Means were compared using Tukey's *post hoc* test. Additionally, contrast analysis was employed to ascertain the differences, if any, between the treatments cPRO and dPRO. Significance was declared at *P* ≤ 0·05.

## Results and discussion

### Nutrient intake and digestibility

Data on food intake and apparent digestibility are presented in Table 1. The palatability of the diet and daily food intake were similar among the groups. The apparent digestibility of DM, crude protein, ether extract and N-free extract was not influenced by the source of probiotics. However, the observed increase (*P* = 0·034) in crude fibre digestibility in the cPRO group could have been due to greater fermentability of the undigested fibre entering the hindgut, possibly due to an elevated population of fibrolytic microbes induced by the canine-origin probiotic *L. johnsonii* CPN23.

**Table 1.** Effect of source of probiotics on the food intake and digestibility of nutrients in Labrador dogs (Mean values with their standard errors; *n* 5)

	Dietary group						
	Control		cPRO		dPRO		
	Mean	se	Mean	se	Mean	se	P*
Palatability†	1·72	0·31	1·68	0·28	1·68	0·16	0·961
Mean daily food (DM) intake							
DM intake (g)	354·4	22·9	361·8	50·2	364·9	20·1	0·883
DM intake (g/kg body weight^0·75^)	34·45	0·05	34·50	0·26	34·44	0·12	0·886
Digestibility of nutrients (g/kg)							
DM	830	23·7	836	40·5	847	11·0	0·622
Organic matter	848	21·3	853	37·1	865	11·5	0·595
Crude protein	796	30·3	816	35·7	826	24·5	0·317
Ether extract	877	7·5	873	10·4	890	5·5	0·341
Crude fibre	354^a^	15·4	419^b^	13·7	386^a,b^	16·4	0·034
N-free extract	904	9·0	901	17·7	914	6·6	0·720

Control, basal diet alone; cPRO, basal diet supplemented with probiotic of canine origin; dPRO, basal diet supplemented with probiotic of dairy origin.

^a,b^ Mean values with unlike superscript letters were significantly different (*P* ≤ 0·05).

* Based on one-way ANOVA.

† Based on a 1–4-point scale.

### Faecal characteristics

Data on faecal characteristics are presented in Table 2. There was no influence of the probiotics on the faecal score or on the frequency of defecation. A significant (*P* = 0·012) reduction in faecal ammonia concentration was evident in both the cPRO and dPRO groups in comparison with the control. Ammonia is formed during colonic fermentation of protein and is considered detrimental to health. Probiotics are presumed to induce lowered production of ammonia. Faecal lactate tended to be higher (*P* = 0·091) in the cPRO group as compared with control. Additionally, contrast analysis also revealed a trend for higher (*P* = 0·059) lactate in the cPRO group compared with the dPRO group. Increased lactate production along with SCFA lowers the pH of the hindgut which, besides facilitating greater protonation of ammonia, leading in turn to its higher faecal excretion^(^^16^^)^, is considered detrimental to pathogenic micro-organisms^(^^17^^)^.

**Table 2.** Effect of source of probiotic on the physical and fermentative indices of faeces of Labrador dogs (Mean values with their standard errors; *n* 5)

	Dietary group						
	Control		cPRO		dPRO		
Attributes	Mean	se	Mean	se	Mean	se	*P*†
Physical characteristics							
Faecal score‡	2·53	0·06	2·76	0·54	2·67	0·15	0·553
Defecation frequency (times/d)	1·85	0·38	1·75	0·35	1·95	0·33	0·679
Faeces voided (g/d)							
Fresh (as is)	225·15	52·54	221·89	42·87	211·89	46·07	0·900
Dry	60·24	9·61	58·24	11·60	55·63	4·15	0·727
Faecal composition (g/kg)							
DM	217·2	36·5	208·5	10·7	213·2	29·7	0·888
Moisture	782·8	36·5	791·5	10·7	786·8	29·7	0·888
Faeces (g/kg DM consumed)							
Wet faeces	639·5	161·8	624·7	145·3	579·1	109·9	0·783
Dry faeces	169·8	23·7	164·1	40·5	152·6	11·0	0·622
Chemical characteristics							
pH	6·26	0·44	6·02	0·19	6·16	0·25	0·480
Ammonia (μmol/g DM)	32·63^b^	3·23	26·45^a^	3·18	27·20^a^	2·36	0·012
Lactate (μmol/g DM)	16·93	3·48	22·13	2·50	19·98	4·11	0·094
SCFA (μmol/g DM)							
Acetate	354·70^a^	41·56	490·41^c^	46·72	428·94^b^	36·34	0·001*
Propionate	199·21^a^	32·78	213·51^a,b^	20·07	244·69^b^	34·53	0·086
Butyrate	73·39^a^	10·14	116·33^c^	13·59	88·20^b^	7·11	<0·0001*
Total SCFA	627·31^a^	81·22	820·26^b^	67·82	761·82^b^	49·83	0·002
BCFA (μmol/g DM)							
Isobutyrate	4·75	1·33	4·25	1·01	3·85	0·80	0·435
Isovalerate	4·23^b^	0·37	3·83^a,b^	0·42	3·09^a^	0·91	0·037
Valerate	14·98^b^	3·40	9·57^a^	1·61	13·21^a,b^	3·86	0·049*
Total BCFA	23·96^b^	3·21	17·65^a^	2·47	20·15^a,b^	3·72	0·026

Control, basal diet alone; cPRO, basal diet supplemented with probiotic of canine origin; dPRO, basal diet supplemented with probiotic of dairy origin; BCFA, branched-chain fatty acids.

^a,b,c^ Mean values with unlike superscript letters were significantly different (*P* ≤ 0·05).

* Significant differences between cPRO and dPRO by contrast analysis.

† Based on one-way ANOVA.

‡ Based on a 1–5-point scale.

Faecal concentrations of both acetate and butyrate were higher (*P* *<* 0·001) in the cPRO group than the dPRO group which, in turn, were higher as compared with control. Additionally, contrast analysis also revealed higher acetate (*P* = 0·019) and butyrate (*P* = 0·001) levels in the cPRO group than the dPRO group, indicative of better adaptation of the canine-origin probiotic in the hindgut of the dogs in comparison with the dairy-origin probiotic. Acceleration in the net production of SCFA and lactic acid by supplementation of probiotics lowers the net production of ammonia, and a similar result was evident in the present study. Faecal levels of isovalerate were lower (*P* = 0·037) in the dPRO group while those of valerate were lower (*P* *<* 0·05) in the cPRO group compared with control. Consequently, faecal levels of total BCFA were reduced (*P* *<* 0·01) in the cPRO group in comparison with control. The BCFA isobutyrate and isovalerate are produced from protein fermentation, specifically from the deamination of valine and leucine, respectively, and are generally believed to be putrefactive leading to production of toxic metabolites deleterious for host health^(^^18^^,^^19^^)^. The present observation of a generalised reduction in BCFA in both the probiotic-supplemented groups, therefore, is indicative of their health-promoting effects. Further, the reduction (*P* *<* 0·05) in the total BCFA in the cPRO group *v*. control is suggestive of the advantages of using the species-specific probiotic in dogs.

### Immune response

Skin induration tended to improve (*P* = 0·053) in the cPRO group (8·3 (se 0·3) mm) group when compared with control (7·5 (se 0·2) mm) while that of the dPRO group (7·9 (se 0·3) mm) was comparable with both. There was, however, no variation in the antibody response against sheep erythrocytes among the three dietary groups. The CMI response, mediated by thymus-derived T-lymphocytes which are responsible for DTH reactions, is considered as a good indicator of the effector phase of the CMI response *in vivo*^(^^20^^)^. The improvements evident in the DTH response by the cPRO group dogs *v*. the control, in turn, imply that the canine-origin probiotic acted better at inducing an augmented CMI response than that of the dairy-origin probiotic. Stimulation of systemic components of the immune system, in particular the CMI, may help to regulate changes in the gut microflora, for example by increasing macrophage phagocytic activity using lactic acid bacteria^(^^21^^)^. Similarly, use of Jerusalem artichoke as a prebiotic has been reported to improve the DTH response to phytohaemagglutinin-P in dogs^(^^22^^)^.

### Conclusion

The results are indicative of the superiority of a canine-origin probiotic over a dairy-origin probiotic, when supplemented in the diet of dogs.

## Supplementary material

For supplementary material accompanying this paper visit http://dx.doi.org/10.1017/jns.2017.35. click here to view supplementary material
