# Supplementary material for: Comparative assessment of canine-origin Lactobacillus johnsonii CPN23 and dairy-origin Lactobacillus acidophillus NCDC 15 for nutrient digestibility, faecal fermentative metabolites and selected gut health indices in dogs
Source: J Nutr Sci. 2017 Jul 31;6:e38. doi: 10.1017/jns.2017.35 (PMC5672308; doi:10.1017/jns.2017.35)
Supplement: Supplementary file 1 [file S2048679017000350sup001.doc]

**Supplementary Table S1.** Composition and nutrient content of the basal diet*† fed to the experimental dogs

| Composition | Level |
| --- | --- |
| Ingredient composition (% as fed basis) |  |
| Rice | 49.8 |
| Bengal gram | 31.3 |
| Soyabean meal | 10.7 |
| Soyabean oil | 5.9 |
| Dicalcium phosphate | 1.3 |
| Calcium carbonate | 1.0 |
| Chemical composition |  |
| Dry matter (DM; %) | 22.11 |
| % DM basis |  |
| Organic matter | 96.20 |
| Crude protein | 22.25 |
| Ether extract | 6.37 |
| Crude fibre | 4.58 |
| N-free extract | 63.00 |
| Crude ash | 3.80 |
| Metabolisable energy‡ (kJ/g) | 14.75 |

*Pressure cooked at 15 psi for 10 min.

†Additionally supplemented with a trace mineral supplement (provided per kg of diet: Mn: 14.2 mg; Fe: 110 mg; Cu: 9 mg; Co: 1.8 mg; Zn: 150 mg; I: 1.6 mg; Se: 0.3 mg) and a vitamin supplement (provided per kg of diet: Vitamin A: 11000 IU; Vitamin D: 910 IU; Vitamin E: 57.5 IU; Vitamin K: 0.65mg; thiamin: 7.56 mg; riboflavin: 11.89 mg; pantothenic acid: 18.50 mg; niacin: 93.16 mg; pyridoxine: 6.60mg; biotin: 12.42 mg; folic acid: 1,142.10 mg; Vitamin B_12_: 164.87 mg).

‡Calculated as per NRC*: ME = 35·564 kJ ME/g fat + 14·644 kJ ME/g CP + 14·644 kJ ME/g nitrogen-free extract.

*National Research Council (2006) *Nutrient Requirements of Dogs and Cats.* Washington, DC: National Academies Press.
